# Kohler Disease With Concurrent Avascular Necrosis of the Medial Cuneiform: A Report of Two Cases

**DOI:** 10.7759/cureus.89411

**Published:** 2025-08-05

**Authors:** Katie W Holley, Benjamin J Price, William F McCormick, Zachary L Littlefield, Brandon M Green

**Affiliations:** 1 Orthopaedic Surgery, East Tennessee State University Quillen College of Medicine, Johnson City, USA

**Keywords:** avascular necrosis (avn), foot, kohler disease, navicular, pediatrics

## Abstract

Two patients, ages five and eight, presented to a pediatric orthopedic surgeon with dorsomedial midfoot pain. After further evaluation, each was diagnosed with idiopathic avascular necrosis of the navicular, also known as Kohler disease, with concurrent avascular necrosis of the ipsilateral medial cuneiform. Both patients were treated conservatively with rest, immobilization, limited weight-bearing, and reduced activity, and both saw resolution of symptoms and radiographic findings by the completion of follow-up. This report aims to reassure physicians that, even with uncommon and unexpected radiographic findings, Kohler disease can be managed conservatively and can result in full recovery and return to activity as soon as 12 weeks after initiating treatment.

## Introduction

Alban Kohler first described Kohler disease in 1908 as idiopathic avascular necrosis of the navicular bone exclusively occurring in the pediatric population [1]. It is one of many osteochondroses, a group of diseases affecting the growing skeleton, typically resulting from vascular pathologies or overuse.

The navicular forms by endochondral ossification with variable timing and pattern, although it is typically the last bone to ossify in the foot. It plays an important role in the midfoot as the primary transmitter of weight from the forefoot to the hindfoot [2]. Articulating with each of the three cuneiforms, it maintains the medial and central columns of the foot, resulting in an extraordinary amount of weight and pressure through the bone. Though not fully understood, Kohler disease affects up to 2% of the pediatric population and is thought to result from compression of the incompletely ossified navicular bone by the adjacent, fully ossified tarsal bones, leading to compromised blood flow in the central third watershed area [3].

Conversely, reports of avascular necrosis of the medial cuneiform have been described since the mid-1900s [4, 5]. It has occasionally occurred in the presence of navicular avascular necrosis in skeletally mature patients secondary to systemic diseases or corticosteroid use [6, 7]; however, this is extraordinarily rare in the presence of idiopathic avascular necrosis of the ipsilateral navicular in a pediatric patient (Kohler disease) [8, 9]. The limited medical literature on this topic indicates a rarity of this occurrence.

Kohler disease patients are typically four to seven years old, presenting with foot pain and point tenderness over the dorsomedial midfoot. Examination will disclose tenderness without associated erythema and pain when stressing midfoot joints [9]. Typically, a self-limited disease, patients are managed conservatively with rest, casting, and limited weight bearing [3]. Rarely, after failure of conservative management, does it require surgical intervention, which is navicular decompression, microcirculation reconstruction, or some combination of the two [10].

## Case presentation

Patient 1

In our first case, a five-year-old male patient presented to the pediatric orthopedic clinic with right foot pain. Our patient had a history of atopy; however, no other significant medical history, surgical history, or family history. The patient reportedly sustained an injury to the right foot while playing basketball two to three weeks prior to initial evaluation and had experienced this pain ever since. He was first seen at a local urgent care, where he was given a diagnosis of Kohler disease, against acute fracture or sprain based on radiographs. He was placed in a walking boot and scheduled for follow-up with a pediatric orthopedic surgeon. 

**Figure 1:**
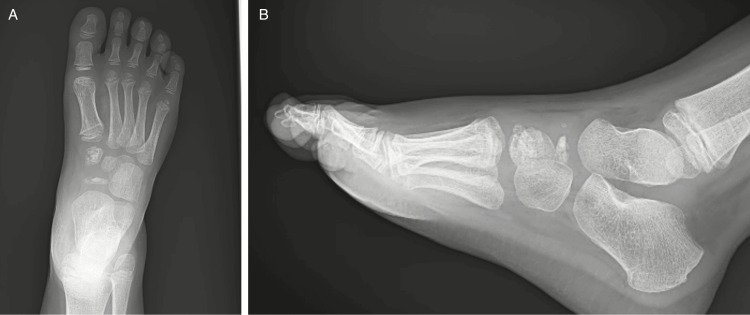
Radiographs from Patient 1’s initial visit, including anteroposterior (A) and lateral (B) views of the right foot of the five-year-old patient; note the “wafer-thin” (i.e., thin, sclerotic) appearance of the navicular, typical of Kohler disease, and the inconsistently fragmented appearance of the medial cuneiform.

At his first visit with the pediatric orthopedic surgeon, a physical exam showed no obvious deformity or ecchymosis present about the midfoot; however, he had notable tenderness to palpation over the navicular bone. The patient was neurovascularly intact with a normal range of motion of the bilateral lower extremities. The patient had difficulty with ambulation due to pain. Repeat radiographs (Figure 1) were obtained of the right foot at that time, which revealed avascular necrosis of the navicular with associated avascular necrosis of the medial cuneiform.

**Figure 2:**
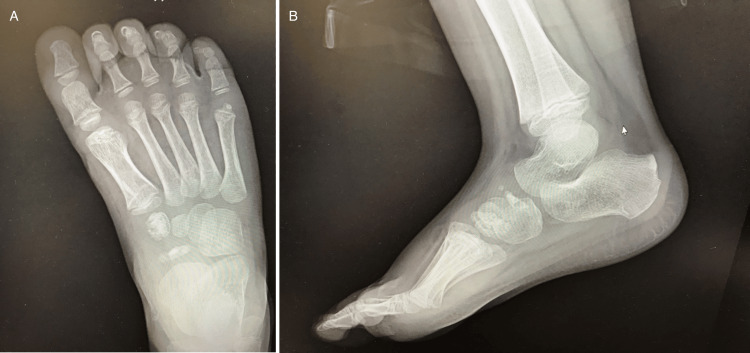
Radiographs obtained 10 weeks following initial diagnosis; anterior-posterior (A) and lateral (B) views of the right foot demonstrate progressive healing of the navicular and medial cuneiform with sclerosis and uniform osseous margins.

A weight-bearing cast was placed on the patient’s right lower extremity, and he was assigned a limited weight-bearing status. An MRI of the right foot was also ordered for further evaluation of the pathology; however, this was not obtained and is not significant for diagnosis or management. At his six-week follow-up appointment, the cast was removed. The patient was then given a walking boot and was allowed to bear full weight as tolerated. At his next follow-up appointment four weeks later, the patient was asymptomatic and pain-free with activity and palpation of his midfoot. He was then released for activity without restrictions. His repeat radiographs 10 weeks after initial evaluation (Figure 2) showed progressive healing with sclerosis and decreased fragmentation of his navicular and medial cuneiform.

Patient 2

Our second patient was an eight-year-old male patient who presented to the pediatric orthopedic clinic concerned with right foot pain for approximately six months. The patient had been limping and walking on the lateral aspect of his foot for the duration of that time. The patient’s mother reported noticing increasing difficulty with ambulation and playing. 

**Figure 3:**
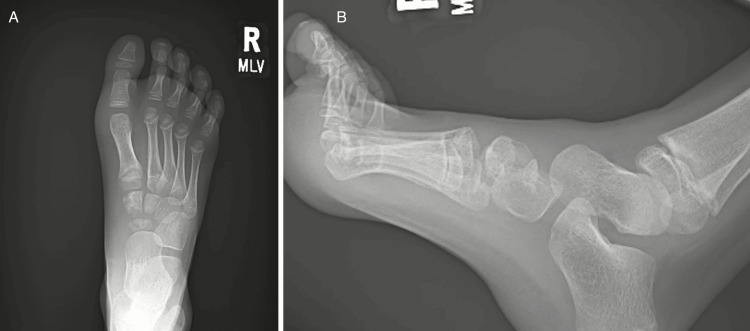
Radiographs from Patient 2’s initial visit, including anteroposterior (A) and lateral (B) views of the right foot of the eight-year-old patient. Loss of the medial margin of both the navicular and medial cuneiform can be seen.

The patient had a past medical history of developmental dysplasia of the right hip, which was treated in 2016 with an adductor tenotomy and closed reduction followed by a Pemberton osteotomy. This history was found to be insignificant, as the patient has had no issues with his right lower extremity following his surgical correction, and Kohler disease is not an associated sequela of hip pathology. On physical exam, the patient was concerned with point tenderness over both the navicular and medial cuneiform. The patient was neurovascularly intact with a normal range of motion of the bilateral lower extremities. Radiographs of the right foot were obtained (Figure 3), demonstrating avascular necrosis of the navicular with associated avascular necrosis of the medial cuneiform.

An MRI of the right foot was obtained for further evaluation and showed no significant additional findings. Following this, the patient was referred to endocrinology for a workup of potentially associated metabolic and endocrine abnormalities. Lab results indicated pre-diabetes and elevated thyroid-stimulating hormone (TSH); however, these were determined to be incidental, transient abnormalities after repeat testing and, therefore, insignificant.

**Figure 4:**
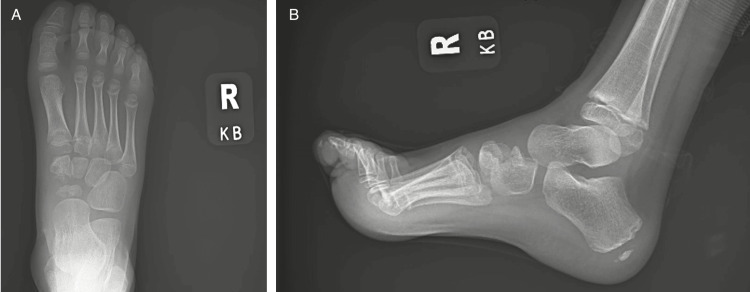
Anterior-posterior (A) and lateral (B) view radiographs were obtained eight weeks after initial diagnosis. Images demonstrate bone healing with uniform sclerosis and progressive ossification of the navicular and medial cuneiform.

The patient was treated conservatively in a walking boot with reduced activity and weight-bearing status. He was re-evaluated at the pediatric orthopedic clinic approximately eight weeks following initial diagnosis, and repeat radiographs were obtained (Figure 4). The patient was accompanied by his mother, who noted resolution of the patient’s pain. He was then made weight-bearing as tolerated and released for activity without restrictions.

## Discussion

Kohler disease is a self-limited disease that often presents in the pediatric population between the ages of four and seven, often following trauma or stress to the navicular bone. Here we have shown that it can rarely present with associated concurrent avascular necrosis of the ipsilateral medial cuneiform. Fortunately, nonoperative management is the literature-supported gold standard for both pathologies [2,3], and both patients have shown significant improvement and resolution of symptoms with temporary immobilization and decreased activity. 

Various cases have been published promoting nonoperative management and its positive outcomes. Multiple case reports have accounted for cases of bilateral navicular avascular necrosis with no mention of complications following conservative treatment [11,12,13]. These cases suggest that even in the presence of more extensive radiographic findings, favorable outcomes can be achieved with conservative management. A series following fourteen patients for an average of over 31 years determined that patients with Kohler disease “can be expected to have a normal foot at adulthood” [14], and prior long-term studies have shown similar success with conservative management [15] and a completely symptom-free cohort after 33 years of follow-up [16].

In the literature, there is no well-defined consensus regarding time to symptom resolution, often ranging from a month to two years; however, radiographic resolution is usually seen within one to three months after initial diagnosis [2, 3]. Alternative management includes microsurgical decompression and revascularization, as reported by one series following three patients who had failed conservative management. All three patients reported resolution of symptoms at three months postoperatively, establishing surgery as an effective alternative. It should be considered, however, that operative management carries the associated risks of surgery as well as foot scars and potential for tendon adhesions [17].

No current reports describe permanent debilitating sequelae of Kohler's disease or avascular necrosis of the medial cuneiform. However, avascular necrosis of the tarsal bones in other populations is not without its complications and demonstrates the probable signs of suboptimal outcomes. Muller-Weiss syndrome, present in the adult population, is well known for its subsequent naviculocuneiform and talonavicular arthritis [18] and can lead to the need to restore the medial column of the foot surgically [19]. Fortunately, none of these outcomes have been seen following appropriate management of Kohler disease; however, patients should be observed for similar signs and symptoms.

## Conclusions

While presenting similarly to isolated idiopathic avascular necrosis of the navicular, radiographic findings of additional necrotic bones in the midfoot can be concerning for the physician, the patient, and parents. In this series, we have demonstrated that, although radiographically unsettling, concurrent avascular necrosis of the navicular and the ipsilateral medial cuneiform should be treated with the standard management for Kohler disease. Both patients described here had similar disease courses, treatment, and recovery as recorded in subsequent follow-up visits. With very few, if any, mentions of these findings in the literature, our goal is to reassure physicians that even with medial cuneiform avascular necrosis, rest, splinting, casting, and limited weight bearing lead to resolution of radiographic findings and symptoms within the usual time course of weeks to months for the management of Kohler disease. Every patient should be treated individually, with careful consideration to rule out more serious underlying pathologies. Further research and the reporting of similar cases will help strengthen the evidence supporting this approach and ensure appropriate care for all patients.
